# Protocadherin-1 is a glucocorticoid-responsive critical regulator of airway epithelial barrier function

**DOI:** 10.1186/s12890-015-0078-z

**Published:** 2015-07-31

**Authors:** Yutaka Kozu, Yasuhiro Gon, Shuichiro Maruoka, Kuroda Kazumichi, Akiko Sekiyama, Hiroyuki Kishi, Yasuyuki Nomura, Minoru Ikeda, Shu Hashimoto

**Affiliations:** Nihon University School of Medicine Division of Respiratory Disease, 30-1 Ohyaguchi-Kamicho, Itabashiku, Tokyo 173-8610 Japan; Nihon University School of Medicine Division of Microbiology, 30-1 Ohyaguchi-Kamicho, Itabashiku, Tokyo 173-8610 Japan; Nihon University School of Medicine Division of Otolaryngology, 30-1 Ohyaguchi-Kamicho, Itabashiku, Tokyo 173-8610 Japan

**Keywords:** *PCDH1*, Bronchial asthma, Chronic rhinosinusitis, Airway barrier function, Corticosteroids, Bronchial hyperresponsiveness, Tight junction, E-cadherin

## Abstract

**Background:**

Impaired epithelial barrier function renders the airway vulnerable to environmental triggers associated with the pathogenesis of bronchial asthma. We investigated the influence of protocadherin-1 (*PCDH1*), a susceptibility gene for bronchial hyperresponsiveness, on airway epithelial barrier function.

**Methods:**

We applied transepithelial electric resistance and dextran permeability testing to evaluate the barrier function of cultured airway epithelial cells. We studied *PCDH1* function by siRNA-mediated knockdown and analyzed nasal or bronchial tissues from 16 patients with chronic rhinosinusitis (CRS) and nine patients with bronchial asthma for *PCDH1* expression.

**Results:**

*PCDH1* was upregulated with the development of epithelial barrier function in cultured airway epithelial cells. Immunocytochemical analysis revealed that *PCDH* localized to cell-cell contact sites and colocalized with E-cadherin at the apical site of airway epithelial cells. *PCDH1* gene knockdown disrupted both tight and adhesion junctions. Immunohistochemical analysis revealed strong *PCDH1* expression in nasal and bronchial epithelial cells; however, expression decreased in inflamed tissues sampled from patients with CRS or bronchial asthma. Dexamethasone (Dex) increased the barrier function of airway epithelial cells and increased *PCDH1* expression. *PCDH1* gene knockdown eradicated the effect of Dex on barrier function.

**Conclusion:**

These results suggest that PCDH1 is important for airway function as a physical barrier, and its dysfunction is involved in the pathogenesis of allergic airway inflammation. We also suggest that glucocorticoids promotes epithelial barrier integrity by inducing *PCDH1*.

## Background

Asthma is a chronic inflammatory disorder of the airways characterized by inflammation, airway hyperresponsiveness, and reversible airflow obstruction [1]. Several cell types have been implicated in the pathogenesis of asthma; airway epithelial barrier dysfunction plays an important role [2]. Therefore, epithelial barrier function, which biologically limits the passage of foreign substances, including inhaled allergens, into the body, plays an important role in airway defense. In general, epithelial barrier function is maintained through a series of cell junctions on the apical side of cells, including tight junctions (TJs) and adherence junctions (AJs) [3].

Bronchial hyperresponsiveness (BHR), the key feature of asthma, is a functional abnormality in which airway constriction is triggered by environmental stimuli that otherwise do not affect healthy individuals. Although airway inflammation is strongly implicated in BHR, the mechanisms underlying BHR remain unclear [4, 5]. Protocadherin-1 (*PCDH1*) was recently identified as a susceptibility gene in asthma [6, 7]. Koppelman et al. performed linkage and mapping analysis in 200 Dutch asthmatic patients with the goal of detecting genes on chromosome 5q31–q33 that are associated with BHR [6]. They found a significant relationship between *PCDH1* and BHR. A follow-up study revealed the same significant relationship between *PCDH1* and BHR in seven of eight populations analyzed (Dutch, English, and American subjects) [6].

PCDH1 belongs to the cadherin protein superfamily and contains a 110-amino acid repeat sequence called the cadherin motif. The cadherin superfamily includes E-cadherin (E-cad), N-cadherin, P-cadherin, desmosomal cadherin, and PCDH [8]. Koning et al. found that *PCDH1* mRNA expression increased during differentiation of cultured airway epithelial cells, which suggested that PCDH1 is important in this process [9].

Formation of the epithelial barrier is an important process during airway epithelial differentiation; however, it is not clear if PCDH1 participates in epithelial barrier formation. In this study, we tested the hypothesis that functional abnormalities due to PCDH1 dysregulation may affect epithelial barrier formation and thereby contribute to the pathogenesis of asthma.

## Methods

### Cells and reagents

Transformed human bronchial epithelial cells (16HBE14_0−_, abbreviated as 16HBE cells [10, 11] and 1HAE_0−_, abbreviated as 1HAE cells [12]) were kindly provided by Prof. Dieter C. Gruenert (Gene Therapy Center, Cardiovascular Research Institute, Department of Laboratory Medicine, University of California). Calu-3 cells, an airway epithelial cell line derived from lung cancer, were obtained from the American Type Culture Collection (Rockville, MD, USA) [13]. Dexamethasone (Dex) and fluorescein isothiocyanate-labeled dextran (FITC-dextran; 4 and 10 kDa) were purchased from Sigma Chemical Company (St. Louis, MO, USA).

### Cell culture

16HBE cells were grown in minimum essential medium (MEM) with 10 % (v/v) fetal bovine serum (FBS). For our experiments, these cells were passaged 20–40 times. Calu-3 cells were maintained in a 1:1 mixture of Ham’s F12 (Gibco Invitrogen Corp., Paisley, UK) and Dulbecco’s Modified Eagle Medium (Sigma), with 10 % FBS (SAFC Biosciences, Lenexa, KS, USA), and passaged 20–40 times before use. 1HAE cells were grown in MEM with 10 % (v/v) FBS and passaged 10–30 times before use.

### siRNA transfection

16HBE cells were grown in six-well plates to 50 % confluence and transfected individually with either 50-nM Silencer Select Control small interfering RNA (siCtlRNA, cat. 12935–112; Invitrogen, Carlsbad, CA, USA) or human *PCDH1* siRNAs (siPCDH1_1, siPCDH1_2, and siPCDH1_3, all obtained from Sigma-Aldrich) for 24 h using Lipofectamine RNAiMAX (Invitrogen), according to the manufacturer’s instructions. The transfected cells were seeded on Transwell chambers (Corning Life Sciences, Corning, NY, USA) before replacing the transfection medium with complete medium with or without Dex.

### RNA extraction and real-time PCR

Total RNA was extracted from 16HBE cells with the RNAiso Reagent (TaKaRa, Japan). First-strand cDNA was synthesized from 2 μg total cellular RNA with the PrimeScript RT reagent Kit (TaKaRa). To amplify *PCDH1*, specific primers were designed based on the gene sequences. Gene-specific primer sets were designed for human *PCDH1* isoforms 1 and 2 as follows**:** PCDH1 isoform 1, 5′**-**GACTCTTCCAGATTGGGTCACAT-3**′** and 5**′**-CTTGCCGCGGTCACTGA-3′**;** PCDH1 isoform 2, 5′**-**TGCCAATGCAGAAATCGAATAC-3′ and 5′-CGGGCCCTGAACAGTGAT-3′. Primers for amplification of GAPDH were used as an internal control: 5′-CAAGTTCAACGGCACAGTCAAG-3′ and 5′-ACATACTCAGCACCAGCATCAC-3′. The Applied Biosystems 7300 Fast Real-Time PCR System with SYBR green PCR master mix (Applied Biosystems) were used according to manufacturer protocols. The reactions were incubated in a 96-well optical plate at 95 °C for 20 s, followed by 40 cycles each of 95 °C for 3 s and 60 °C for 30 s. The threshold cycle (Ct) data were obtained using default threshold settings. Ct is defined as the fractional cycle number at which the fluorescence passes the fixed threshold.

### Measurement of transepithelial electrical resistance

16HBE and 1HAE cells were seeded onto Transwell inserts (Costar, New York, NY, USA) at a density of 2 × 10^5^ cells/cm^2^. Calu-3 cells were seeded onto Transwell inserts at a density of 1 × 10^5^ cells/cm^2^. Cell layer integrity was evaluated by measuring transepithelial electrical resistance (TER) with Millicell-ERS equipment (Millipore Co., Bedford, MA, USA).

### Paracellular FITC-dextran fluxes

The permeability of cell monolayers was determined by FITC-dextran fluxes across the cell layer. A solution containing FITC-dextran of 4 or 10 kDa (1 mg/ml) was added to the apical compartment. Samples (100 μl) were removed from the basal compartments 60 min after addition of FITC-dextran and measured by a PTI fluorometer at 492 nm (excitation) and 520 nm (emission).

### Apoptosis assay

The Annexin V/FITC and propidium iodide (PI) apoptosis detection kit (Becton-Dickinson, Franklin Lakes, NJ, USA) was used to quantitatively measure the phosphatidylserine in apoptotic cells. Briefly, transfected cells (siCtl, siPCDH1) (5 × 10^5^ per well) were seeded into 6-well plates. After 24 h, the cells were harvested and washed three times with ice-cold phosphate-buffered saline (PBS) (pH 7.2). After washing, each sample was centrifuged at 1300 rpm for 3 min at 4 °C. Annexin V/FITC and PI double-staining were performed according to manufacturer instructions. Apoptosis was analyzed on a FACScan flow cytometer (Becton-Dickinson, Heidelberg, Germany) and Annexin V-positive, PI-negative cells were scored as apoptotic (Fig 4). Double-stained cells were considered to be necrotic or late apoptotic.

### Immunofluorescence microscopy

After the indicated culture period on Transwells, cells were fixed with 4 % paraformaldehyde for 30 min at 37 °C. Anti-human ZO-1 mAb (Zymed Laboratories Inc., San Francisco, CA), anti-human E-cad rabbit mAb (Cell Signaling Technology, MA), or anti-human PCDH1 mAb (Santa Cruz) was used as a primary antibody, and Alexa 488-conjugated anti-mouse IgG was used as a secondary antibody. An FV1000-D laser scanning confocal microscope with a 60× objective lens was used to investigate expression.

### Patients

Sixteen patients with chronic rhinosinusitis (CRS) and nine asthmatic patients were enrolled. Tissue samples were obtained during surgical biopsies from the patients. CRS and asthma were defined by the criteria established by the American Academy of Otolaryngology–Head and Neck Surgery Chronic Rhinosinusitis Task Force [14] and the Global Initiative for Asthma guidelines [1], respectively. The subjects’ clinical characteristics are shown in Tables 1 and 2. All ethmoid sinus tissues were collected during surgery to remove nasal polyps. We used nasal tissues from the patients who underwent surgery for nasal septal deviation as a control for CRS. Five lung tissue samples from patients with asthma were collected during surgery for pneumothorax, one sample was collected by pneumonectomy for lung cancer, and three samples were collected during autopsy. We used lung tissues from the patients who underwent surgery for pneumothorax or lung cancer as a control for asthma. The study was approved by the Nihon University Itabashi Hospital Ethics Committee, and written informed consent was obtained from all patients.

**Table 1:** Clinical characteristics of patients with chronic rhinosinusitis (CRS)

	non-CRS (*n* = 9)	CRS (*n* = 16)	*p*-value
Sex (M/F)	6/3	10/6	
Age (years), median (range)	57.7 (19–82)	57 (24–70)	0.145073
Blood eosinophil (/μl)^a^	122	345	0.030078
Smoking (%)	44.4	33.3	

^a^Data are expressed as mean values

**Table 2:** Clinical characteristics of patients with asthma

	Nonasthma (*n* = 9)	Asthma (*n* = 9)	*p*-value
Sex (M/F)	9/0	8/1	
Age (years), median (range)	34.8	45.6	0.114272
FEV1.0 (% predicted)^a^		59.3	
FVC (% predicted)^a^		65.1	
Blood eosinophil (/μl)^a^	173	518	0.006926
Smoking (%)	66.6	33.3	

^a^Data are expressed as mean values

### Western blotting

Stimulated cells were washed twice with ice-cold PBS and lysed in Tris-buffered saline containing 1 % Nonidet P-40, 60 mM octyl-β-glucoside, 2 mM phenylmethylsulfonylfluoride, 10 μg/ml aprotinin, 2 μg/ml leupeptin and pepstatin A, 50 mM NaF, and 1 mM sodium orthovanadate for 30 min on ice. The lysates or immunoprecipitates were centrifuged for 15 min at 14000 *g*. The samples for polyacrylamide gel electrophoresis (PAGE) analysis were mixed with 4× XT sample buffer (Bio-Rad, Hercules, CA) and boiled for 4 min and separated on 10 % sodium dodecylsulfate-PAGE and transferred onto an Immobilon-P membrane (Millipore, Bedford, MA). The membrane was incubated with anti-human ZO-1 mAb (Zymed), anti-human OCLN rabbit mAb (Zymed), anti-human E-cad rabbit mAb (Cell Signaling), and anti-human PCDH1 mAb (Santa Cruz) as a primary antibody and an appropriate secondary horseradish peroxidase-conjugated antibody (Fig. 6). Signals were detected using enhanced chemiluminescence (GE Healthcare, Little Chalfont, UK).

### Immunohistochemistry

We focused on PCDH1 expression in ciliated airway epithelial cells (CECs) from the noninflamed region (NR), where there are few infiltrated inflammatory cells and CECs are histologically intact. We also examined inflamed regions (IR) where inflammatory cells such as eosinophils and lymphocytes had infiltrated the submucosa and where histology indicated that the CECs had sustained damage such as partially shed epithelium or separation of cell junctions. The lung and nasal tissues were paraffin embedded and then cut into sections. These sections were deparaffinized and rehydrated. After antigen retrieval, the endogenous peroxidase was inactivated by 3 % hydrogen peroxide in methanol. Then, these sections were incubated with the primary antibody against PCDH1 (1:500) at room temperature for 1 h for 30 °C at room temperature. Staining was performed with 3,3′-diaminobenzidine, and counterstaining was performed using hematoxylin.

Sections were dehydrated in absolute ethanol and dehydrated in an absolute ethanol series and xylene. After mounting, the sections were observed by light microscopy. The primary antibody was replaced with phosphate-buffered saline (PBS) in the negative controls. All sections were scored in a semiquantitative manner by considering the intensity of cell staining. Intensities were classified as 0 (no staining), +1 (weak staining), +2 (distinct staining), and +3 (very strong staining).

### Statistical analysis

Normally distributed data were expressed as means ± standard errors, and differences between groups were analyzed by Student’s *t*-test. Where not normally distributed, data were summarized using the median and interquartile range and were evaluated by nonparametric Wilcoxon rank sum or Mann–Whitney *U* test. All data were analyzed by Prism (GraphPad Software, La Jolla, CA).

## Results

### Expression of PCDH1 in airway epithelial barrier development

**Fig. 1:**
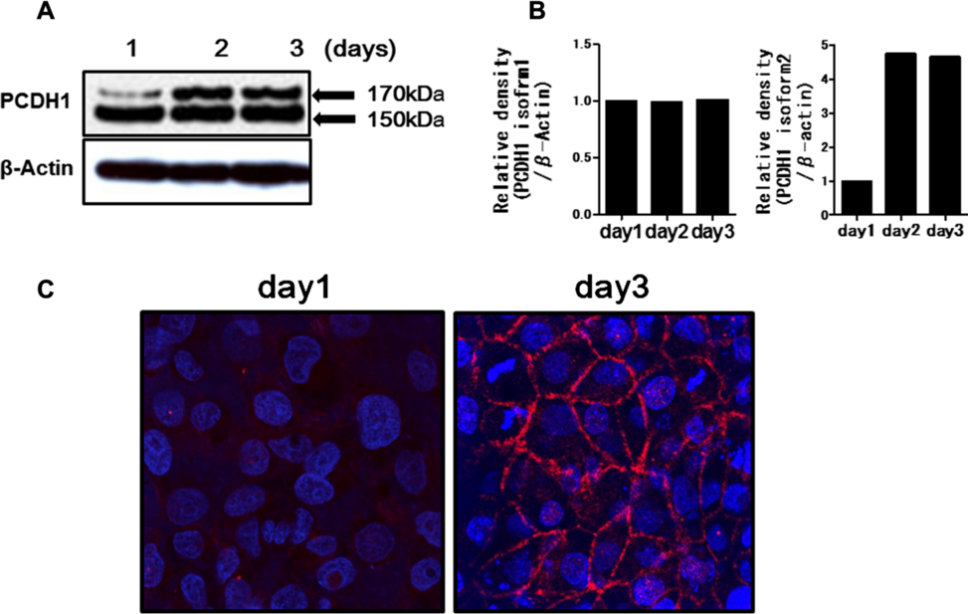
Expression of protocadherin-1 (PCDH1) protein in human bronchial epithelial (16HBE) cells. **a** Time course of PCDH1 protein expression in 16HBE cells. Cell lysates were harvested at the indicated time points. Western blot with anti-PCDH1 antibody showed two bands (150 and 170 kDa) corresponding to PCDH1 isoforms 1 and 2, respectively. β-Actin was used as an internal standard. **b** Graphs showing densitometric quantification of the PCDH1 isoform 1 or 2 bands on western blots, relative to β-actin. **c** Immunocytochemical analysis of PCDH1 (red) and cellular nuclei (blue) in 16HBE cells cultured for 24 and 72 h. The data represent the mean of three independent experiments

The biological roles of PCDH1 in airway epithelium were investigated using polarized 16HBE human airway epithelial cell monolayers. When grown on Transwell filters, 16HBE cells spontaneously polarized and formed TJs [15, 16]. As we previously reported, TER increases steadily and reaches a maximum after 3–5 days [15, 16]. We examined expression of PCDH1 protein during the development of TER in 16HBE monolayers. Consistent with a previous report, PCDH1 protein was detected as two bands, a 170-kDa band that represents the full-length protein (isoform 2) and a 150-kDa band that represents the alternative splicing isoform 1, which lacks exon 2 and thus has no cytoplasmic domain [7]. As shown in Fig. 1a, expression levels of PCDH1 isoform 1 were unchanged over the course of 3 days. However, the expression of PCDH1 isoform 2 markedly increased at days 2 and 3 and overlapped with the increase in TER.We next performed immunocytochemistry to determine PCDH1 localization in 16HBE airway epithelial cells. PCDH1 expression was observed at the sites of cell-cell contacts on day 3 but was undetectable at these sites on day 1 (Fig. 1b).

### siRNA silencing of *PCDH1* impairs 16HBE airway epithelial barrier formation

**Fig. 2:**
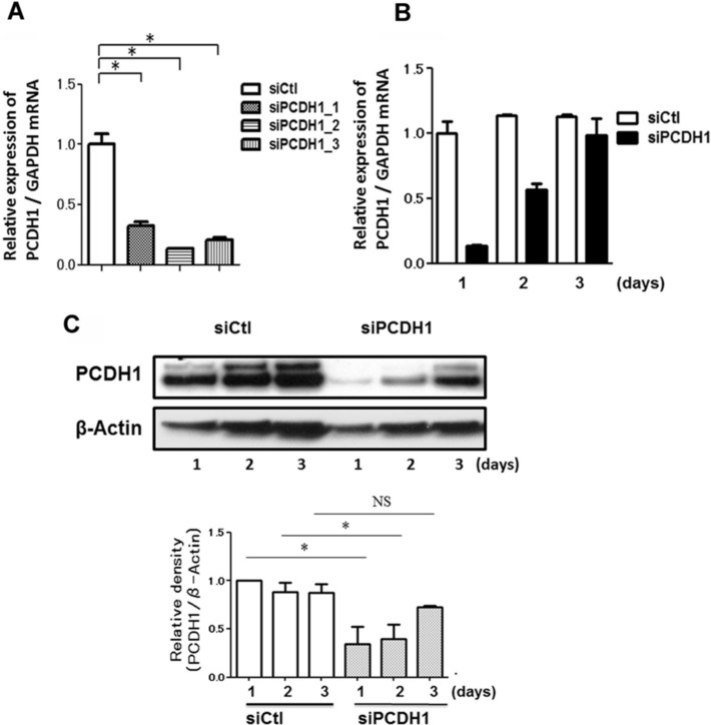
Gene knockdown efficacy of protocadherin-1 (PCDH1)-specific siRNAs. Quantification of PCDH1 mRNA by real-time polymerase chain reaction. **a** mRNA was purified from the cells harvested at 24 h after transfection of the control (siCtl) or PCDH1-specific siRNAs (siPCDH1_1, siPCDH1_2, and siPCDH1_3). Results are expressed relative to the control value (siCtl-treated cells) and are mean ± SD values; *n* = 3 independent samples. Asterisks indicate a statistically significant difference (*p* ≤ 0.05) in the result between that of cells treated with siCtl. **b** Time course of PCDH1 mRNA expression after PCDH1 siRNA transfection. mRNA was purified from cells harvested at indicated time points after the transfection of siCtl or siPCDH1_1. Results are expressed relative to the control value (siCtl-treated cells at day 1) and are mean ± SD values; *n* = 3 independent samples. **c** Time course of PCDH1 protein expression after PCDH1 siRNA transfection. Cell lysates were harvested at the indicated time points after siCtl or siPCDH1_1 transfection and western blotted with anti-PCDH1 antibody. The data represent the mean from three independent experiments (upper photograph). The lower graph shows densitometric quantification of PCDH1 bands on western blots, relative to β-actin. Results are expressed as a relative density compared to the control value (siCtl-treated cells) and are mean ± SD values; *n* = 3 independent samples. Asterisk indicates a statistically significant difference (*p* ≤ 0.05) in the result between that of cells treated with siCtl. NS: not significant

To investigate the roles of PCDH1 in epithelial barrier formation, we specifically depleted PCDH1 by siRNA-mediated silencing. For these experiments, subconfluent 16HBE cells were transfected with siRNAs and then added to Transwell filters at higher density to minimize the influence of cell growth and accelerate epithelial polarization. *PCDH1* mRNA was reduced by > 70 % with one of the three independent siRNAs (siPCDH1_1, siPCDH1_2, and siPCDH1_3) 24 h after addition to Transwell filters (Fig. 2a). Both *PCDH1* mRNA (Fig. 2b) and protein levels (Fig. 2c,d) were transiently suppressed between 24 and 48 h, after which PCDH1 levels slowly began to increase.

**Fig. 3:**
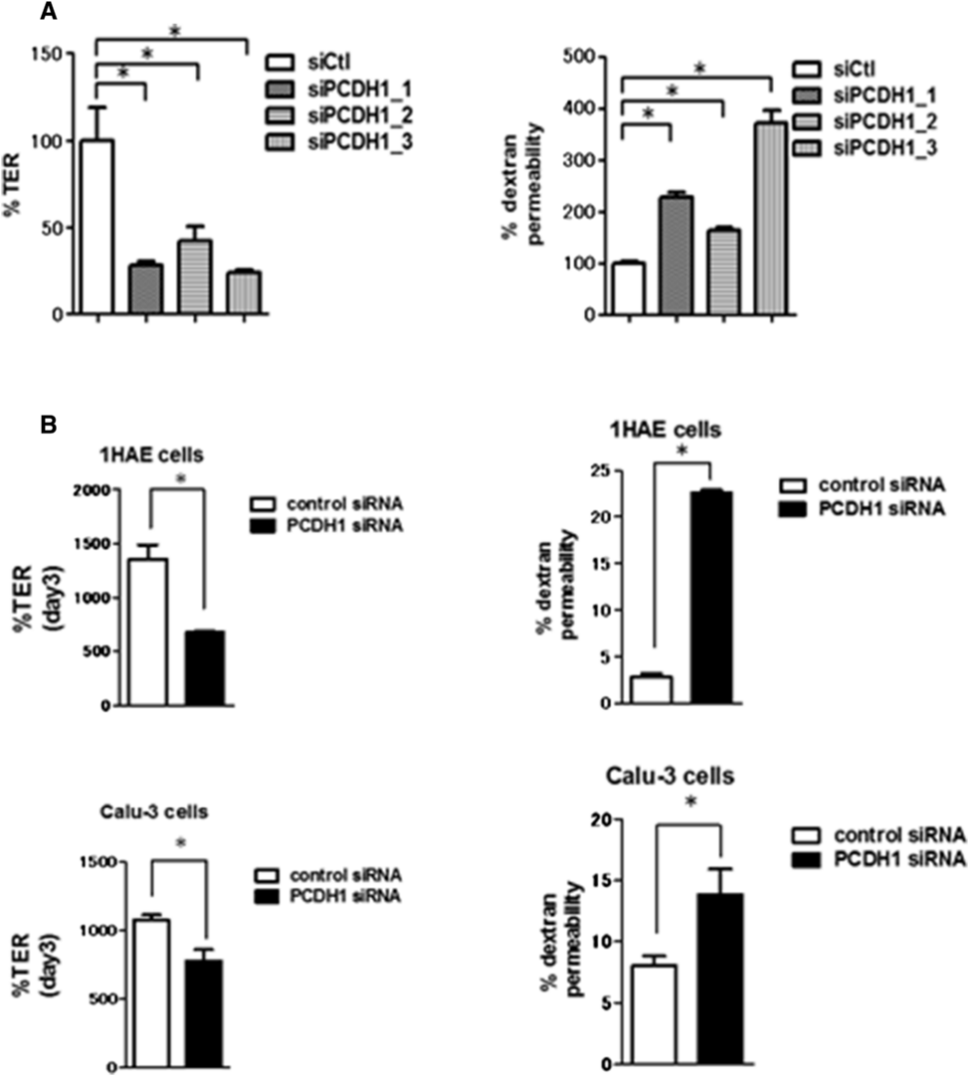
siRNA knockdown of protocadherin-1 (*PCDH1*) impairs epithelial barrier formation. Effect of *PCDH1* knockdown on barrier development. Human bronchial epithelial (16HBE) cells were transfected with control (siCtl) or *PCDH1*-specific siRNAs (siPCDH1_1, siPCDH1_2, and siPCDH1_3). After 24 h, cells were seeded onto the Transwell inserts, and transepithelial electrical resistance (**a**. left) and dextran permeability (**a**. right) were measured at day 3. Results are expressed as a percentage of the control value (siCtl-treated cells) and are mean ± SD values; *n* = 3 independent samples. Asterisk indicates a statistically significant difference (*p* ≤ 0.05) in the result between that of cells treated with siCtl. 1HAE and Calu-3 cells were transfected with control (siCtl) or PCDH1-specific siRNAs (siPCDH1_1). After 24 h, cells were seeded onto the Transwell inserts, and TER (**b**. left) and dextran permeability (**b**. right) were measured at day 3. Results are expressed as a percentage of the control value (siCtl-treated cells) and are mean ± SD (*n* = 3). Asterisks indicate a statistically significant difference (*P* ≤ 0.05) compared to cells treated with siCtl

Over the 3-day period, TER in the *PCDH1*-knockdown 16HBE cell monolayers was approximately 60 % lower than that in the control cell monolayers (Fig. 3a. left). TER is influenced by paracellular and/or intracellular flux of ions [17]. Measurement of the permeability of *PCDH1*-knockdown 16HBE cell monolayers with the nonionic macromolecular tracer, FITC-dextran, which can only pass through the cell monolayer via the paracellular route, was examined (Fig. 3a. right). At day 3, the permeability of siPCDH1_1-transfected cell monolayers evaluated by FITC-dextran influx assay was increased relative to that of siCtl-transfected 16HBE cell monolayers. Similar effects were observed in two different airway epithelial cell lines, Calu-3 and 1-HAE, (Fig. 3b).

### Silencing of *PCDH1* does not affect cell growth or viability

**Fig. 4:**
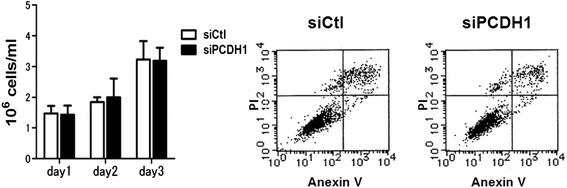
Silencing of PCDH1 does not affect cell growth or apoptosis. 16HBE cells were transfected with control (siCtl) or PCDH1-specific siRNA (siPCDH1_1). Results are mean ± SD. (*n* = 3). (left) The number of live cells was counted daily for 3 days using trypan blue. (right) Apoptosis was detected by Annexin V/PI staining at day 3 after transfection. The x-axis shows AnnexinV-FITC binding and the y-axis pertains to the results for staining with the vital dye propidium iodide. Cells in the lower left quadrant are viable, cells in the lower right are apoptotic, and those in the upper right are late stage apoptotic/dead cells

We next determined if the reduced TER in *PCDH1*-knockdown 16HBE monolayers was caused by reduced cell proliferation or increased apoptosis. Proliferation assays revealed that there were similar cell numbers in *PCDH1*-knockdown and control 16HBE monolayers (Fig. 4. left). Annexin V and propidium iodide staining revealed that *PCDH1* knockdown did not lead to apoptotic cell death (Fig. 4. right). These results indicated that neither decreased cell proliferation nor reduced cell viability accounted for the defective epithelial barrier function in PCDH1-depleted monolayers.

### *PCDH1* knockdown in airway epithelial cells inhibits formation of intercellular junctions

**Fig. 5:**
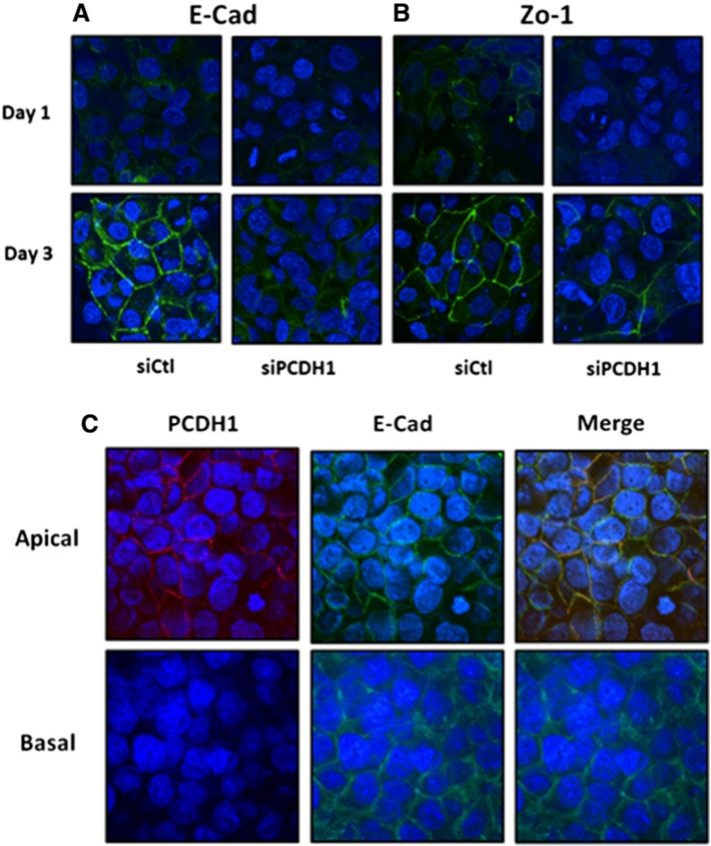
Protocadherin-1 (*PCDH1*) knockdown in airway epithelial cells inhibits the formation of adherence junctions and tight junctions. Immunocytochemical analysis of E-cadherin (E-cad) (**a**) and zonula occludens-1 (**b**) in *PCDH1*-knockdown cells. Human bronchial epithelial (16HBE) cells were transfected with control (siCtl) and *PCDH1*-specific siRNA (siPCDH1_1). After 48 h, cells were subjected to immunocytochemical analysis with antibodies specific to E-cad (green) or PCDH1 (green). The images represent data from three independent experiments. **a** Colocalization of PCDH1 and E-cad. 16HBE cells were cultured for 3 days and stained with anti-PCDH1 and anti- E-cad antibodies. Expression of PCDH1 was mainly co-localized with E-cad at the apical site of cell junctions (upper panel); however, the expression level of PCDH1 was relatively low (lower panel). The images represent data from three independent experiments (**c**)

**Fig. 6:**
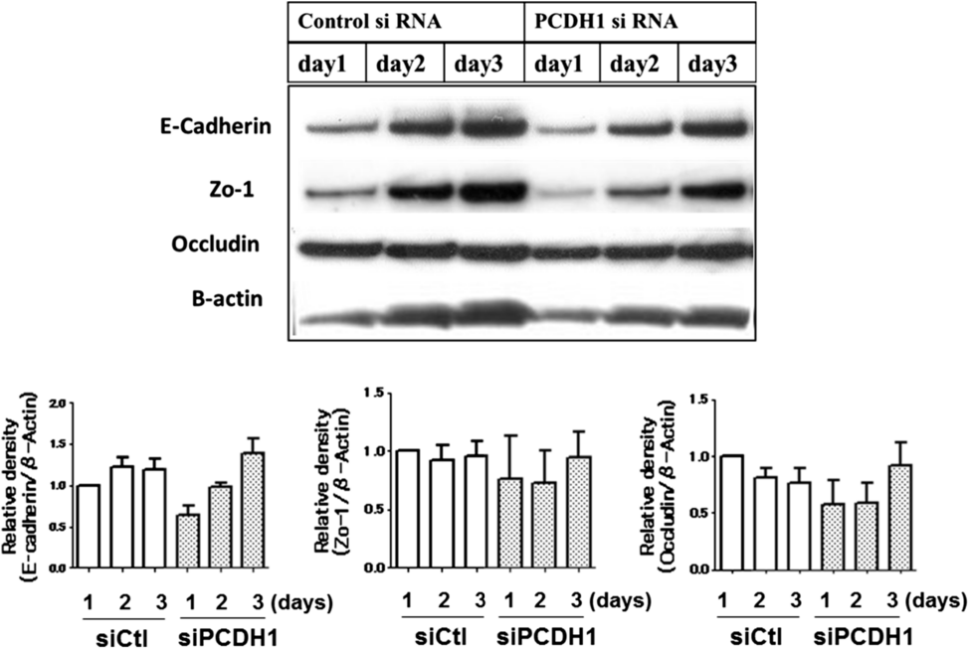
Time course of E-cadherin, ZO-1, and occludin protein expression after PCDH1 knockdown. Cell lysates were harvested at the indicated time points after the transfection of siCtl or siPCDH1_1 and western blotted with anti-PCDH1 antibody. Lower graph shows densitometric quantification of E-cadherin, ZO-1, and occludin bands on western blots, relative to β-actin

To evaluate the role of PCDH1 on epithelial barrier formation, we assessed formation of AJs and TJs in *PCDH1*-silenced and control 16HBE cell monolayers by confocal immunofluorescence microscopy. After 3 days, cells were fixed and stained for AJs and TJs with anti-E-cad-specific antibodies and anti-zonula occludens-1 (ZO-1) antibodies, respectively. E-cad and ZO-1 were strongly expressed and localized as junction proteins at the cell-cell contact sites in the control cell monolayers (Fig. 5a,b, left). In contrast, in *PCDH1*-knockdown monolayers, most of the cells showed substantial reduction of E-cad and ZO-1 staining at the apical surface of cell-cell contact sites (Fig. 5a,b, right). Western blotting revealed similar expression of E-cad, ZO-1, or occludin (OCLN) in the cytosol of *PCDH1*-knockdown and control cells after 3 days (Fig. 6). We compared the localization of PCDH1 and E-cad protein in 3-day cultures of 16HBE cells and found that although PCDH1 expression was low and was mainly co-localized with E-cad at the apical site of cell junctions (Fig. 5).

### Dex enhances epithelial barrier integrity via induction of *PCDH1*

**Fig. 7:**
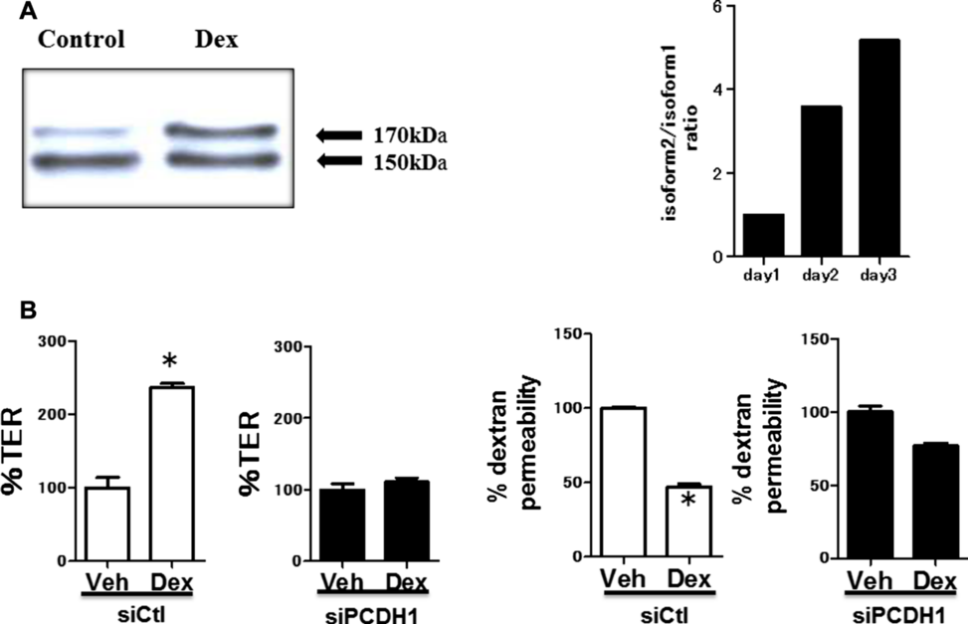
Role of protocadherin-1 (PCDH1) in dexamethasone (Dex)-mediated epithelial barrier integrity. **b** Human bronchial epithelial (16HBE) cells were cultured on Transwell inserts with or without Dex. The cell lysates were harvested 72 h after the culture. Western blot with the anti-PCDH1 antibody showed 150- and 170-kDa bands, corresponding to PCDH1 isoforms 1 and 2, respectively. The graph on the right shows the relative expression of PCDH1 isoforms 1 and 2 on western blots quantified by densitometry. The image and graphs represent data from two independent experiments. **b** PCDH1 knockdown eradicated the enhanced effect of Dex on the development of transepithelial electrical resistance (left) and reduction of dextran permeability (right) in 16HBE cells. Results are expressed as a percentage of the control value (siCtl-treated cells) and are mean ± SD values; *n* = 3 independent experiments. Asterisks indicate a statistically significant difference (*p* ≤ 0.05) in the result between that of cells treated with siCtl

We previously demonstrated that glucocorticoids strongly enhance epithelial barrier integrity in 16HBE airway epithelial cells [15]. Therefore, we determined if PCDH1 was involved in the increased epithelial barrier integrity afforded by the glucocorticoid Dex. Western blotting revealed that Dex increased expression of PCDH1 isoform 2 in 16HBE cells. However, the expression of PCDH1 isoform 1 was not altered by Dex (Fig. 7a). Real-time polymerase chain reaction (RT-PCR) revealed an increase in PCDH1 isoform 2 transcripts in 16HBE cells cultured with Dex (Fig. 7b), whereas the level of PCDH1 isoform 1 did not change (Fig. 7b). Furthermore, *PCDH1* knockdown in 16HBE cells inhibited Dex-dependent increases in TER and alleviated the suppressive effect of Dex on dextran flux (Fig. 7c).

### Expression of PCDH1 in CRS nasal epithelium and asthma airway epithelium

**Fig. 8:**
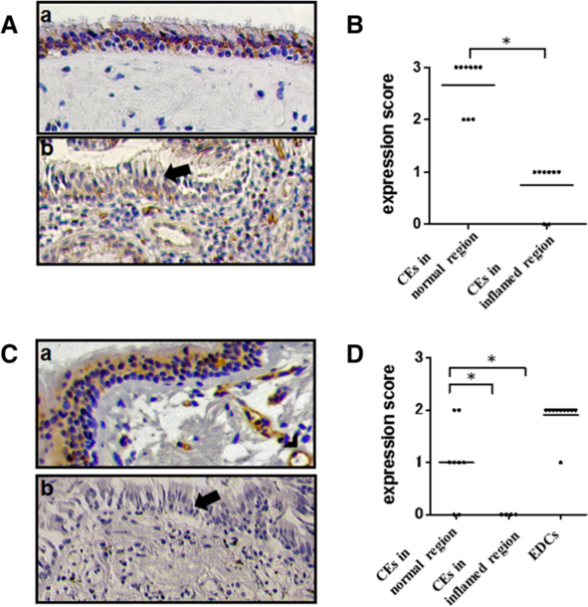
Protocadherin-1 (PCDH1) expression in the nasal tissues of patients with chronic rhinosinusitis (CRS) and the airway of asthmatic patients. **a** Representative image of PCDH1 expression in the nasal tissue from a patient with CRS (a,b). PCDH1 expression of normal region (a) and inflamed region (b) in the nasal tissues of CRS. Arrow shows ciliated airway epithelial cells (CECs) in the inflamed region. **b**. Immunohistochemistry score for PCDH1 expression in CECs of patients with CRS (*n* = 16). The graph shows the expression level of PCDH1 in the normal region and inflamed region. The horizontal bars represent the mean values of the expression score. An asterisk indicates a statistically significant difference (p ≤ 0.05). **c** A representative image of PCDH1 expression in the airway of a patient with asthma (a,b). Normal region (a) and inflamed region (b) in the airway tissues of an individual with asthma. Arrow shows asthma in the inflamed region. Arrowhead shows endothelial cells. **d** Immunohistochemistry score for PCDH1 expression of CECs in asthmatic patients (*n* = 9). The graph shows the expression levels of PCDH1 in the normal region and inflamed region of the lung tissues. EDC stands for endothelial cells. Horizontal bars represent mean value expression scores. Asterisks indicate a statistically significant difference (*p* ≤ 0.05)

To study the expression of PCDH1 in the nasal epithelia of patients with CRS, we performed immunohistochemical staining with anti-PCDH1 antibodies. As shown in Figure 8a, PCDH1 expression in the nasal mucosa was mainly observed in CECs and basal cells. There are no differences in the expression levels of PCDH1 between normal mucosa in control and NR in CRS. In the nasal tissues of CRS, PCDH1 expression was observed in CECs in the NR but not in the IR, as reflected by a significantly higher (*p* = 0.0002) mean staining score (Fig. 8a,b). As shown in Figure 8c, PCDH1 was expressed in CECs from the NR of asthmatic airways and in endothelial cells in the normal region of asthmatic and control airways but was very low or absent in CECs from the IR of asthmatic airways. There are no differences in the expression levels of PCDH1 between normal mucosa in control and NR in asthma. The difference in PCDH1 expression between the two compartments was statistically significant (*p* = 0.034; Fig. 8d).

## Discussion

To our knowledge, this study is the first to demonstrate that *PCDH1*, which has been identified as an airway hyperreactivity-susceptibility gene, has an important role in the formation and maintenance of the intercellular junctions that comprise the airway epithelial barrier. Epithelial barrier dysfunction contributes to the pathogenesis and development of bronchial asthma and CRS [18]. Our study suggests that reduced PCDH1 expression and function is related to the pathogenesis of allergic airway inflammation in bronchial asthma and CRS.

Epithelial barrier function is maintained by TJs and AJs. AJ formation is considered to be especially important in epithelial polarization, which in turn facilitates TJ formation. TJs are required to restrict the nonselective passage of small molecules between cells once contacts have been formed. TJs connect cells firmly to each other by OCLN and members of the claudin family of transmembrane proteins [19]. TJs are linked to the actin cytoskeleton through complexes containing various intracellular proteins such as ZO-1, ZO-2, and ZO-3 [20]. AJs are composed of a transmembrane protein, E-cad, which is linked indirectly to actin through several proteins, including β-catenin [21]. TJ formation is closely related to epithelial cell polarization and requires AJ formation [16].

TJ structures in the airway of asthmatic patients are disrupted [22]. Furthermore, primary cultured airway epithelial cells obtained from asthmatic patients exhibit immature barrier function in differentiation in vitro and are more prone to damage from cigarette smoke than are healthy individuals [23]. Epithelial barrier development in asthmatic patients may be impaired by genetic factors, virus infection, inhaled allergens, or air pollution [22, 24].

PCDH1 co-localized with E-cad at the apical surface of the epithelial cell monolayer, and *PCDH1* knockdown reduced both TJ formation and AJ formation in intercellular spaces at the apical surface in immortalized normal human airway epithelial cell lines (Fig. 7 and Fig. 3b). This suggests that PCDH1 facilitates E-cad assembly, and its loss would inhibit TJ formation through direct or indirect mechanism. Consistent with this, proliferation, apoptosis, or E-cad total levels were not affected by *PCDH1* knockdown (Fig. 5). A limitation of our study is that we did not study the effect of *PCDH1* knockdown on primary cultured epithelial cells because of technical difficulties in achieving sustained siRNA delivery into primary cells.

Local administration of glucocorticoids is the most effective current therapy for bronchial asthma. Recently, we reported that glucocorticoids ameliorated the conditions associated with impaired airway epithelial cell barrier function. Here, we found that the addition of Dex strongly induced expression of PCDH1 isoform-2. This isoform has a long cytoplasmic region, which suggests that it possesses outside-in-signal transduction functionality. Conversely, isoform 1 has no long cytoplasmic region and only has an extracellular region, which suggests that its function differs from that of isoform 2. We also found that the increase in epithelial barrier function was accompanied by an increase in the expression of isoform 2 relative to that of isoform 1. Together, these data suggest that PCDH1 isoform 2 has a positive effect on epithelial barrier formation and that the integrity of barrier function and elevated PCDH1 expression caused by glucocorticoids are probably functionally related events. We will focus on the differences between the functions of individual PCDH1 variants in future studies.

To clarify the contribution of PCDH1 to the pathogenesis of bronchial asthma and CRS, we analyzed the distribution of PCDH1 expression in the airways and nasal mucosal tissue obtained from patients with bronchial asthma and CRS. Human nasal and airway mucosal epithelium mainly is composed of basal cells and CECs. The histological appearance of these epithelia is similar. Here, PCDH1 was mainly expressed in CECs in the airway or nasal epithelium. Considering that the biological barrier function of the epithelium is especially important in the airway, which is constantly in contact with foreign substances, it is not surprising that PCDH1 is highly expressed in the mucosal epithelium of these tissues. We could not compare the expression levels of PCDH1 and other TJs/AJs proteins. But, interestingly, in both asthma and CRS patients, low PCDH1 expression levels in the airway epithelium were observed in regions containing inflammatory cells, large-scale epithelial detachment, and widened intercellular spaces. This suggests that the low PCDH1 expression in this area is associated with increased damage and vulnerability of the epithelial barrier function. We used several sources of lung sections, including autopsy samples obtained from fatal asthmatic patients. All patients were relatively severe asthmatic patients with airflow limitation. In future studies, it will be important to study the expression and function of *PCDH1* in larger numbers of subjects with varying asthma severity.

It is likely that PCDH1, which has been identified as an airway hyperreactivity-susceptible gene, plays an important role in epithelial barrier function. In addition, the results of this study make it clear that expression of this type of gene can be induced by glucocorticoids, which are the most effective agents for bronchial asthma. These results improve our understanding of the relationship between epithelial barrier function and allergic airway inflammation. We have demonstrated that the regulation of epithelial barrier function is mediated through PCDH1 and shown that PCDH1 is downregulated in allergic inflammation. Together, these results suggest that restoration of PCDH1 levels and/or function should be a potential therapeutic strategy for the treatment of bronchial asthma or CRS. Thus, the results of our study may contribute to the development of new treatment methods for allergic airway inflammation.

## Conclusions

These results suggest that PCDH1 is important for airway function as a physical barrier, and its dysfunction is involved in the pathogenesis of allergic airway inflammation. We also suggest that glucocorticoids promotes epithelial barrier integrity by inducing *PCDH1*.

## Abbreviations

BHR: Bronchial hyperresponsiveness
OCLN: Occludin
ZO-1: Zonula occludens-1
TJs: Tight junctions
E-cad: E-cadherin
PCDH1: Protocadherin-1
Dex: Dexamethasone
MEM: Minimum essential medium
FITC-dextran: Fluorescein isothiocyanate-labeled dextran
FBS: Fetal bovine serum
Ct: Threshold cycle
TER: Transepithelial electrical resistance
PBS: Phosphate-buffered saline
CRS: Chronic rhinosinusitis
AJs: Adherence junctions
TJs: Tight junctions
CECs: Ciliated airway epithelial cells
EDCs: Endothelial cells
